# New Frontiers for Molecular Pathology

**DOI:** 10.3389/fmed.2019.00284

**Published:** 2019-12-04

**Authors:** Joanna Domagala-Kulawik

**Affiliations:** Department of Internal Medicine, Pulmonary Diseases and Allergy Medical University of Warsaw, Warsaw, Poland

**Keywords:** lung cancer, immunohistochemistry, molecular pathology, EGFR, molecular testing, immunotherapy, PD-L1, TME

## Abstract

Lung cancer remains a serious oncological problem worldwide. The delayed diagnosis and a prevalence of advanced stages in up to 70% of cases at recognition are still observed. Thanks to targeted therapies and immunotherapy a significant progress in achieving prolonged survival in some lung cancer patients is reported. A precise histopathological diagnosis, especially the recognition of adenocarcinoma, and a progress in the methods of clinical staging underlie the proper qualification of patients for a tailored therapy. The deep molecular characteristics of lung cancer in liquid biopsy, for example blood, bronchoalveolar lavage fluid (BALF), cell suspension from needle aspirates, are currently available. The molecular characteristic has recently been extended with molecular aberrations of BRAF, KRAS, MET, ERBB2, RET, NTRK next to the well-known EGFR mutations and ALK, ROS-1 relocation. The present paper discusses the usefulness of adequate pathological methods and molecular testing for the identification of a broad spectrum of predictive biomarkers for a molecular-directed lung cancer therapy. Immunotherapy with immune checkpoint inhibitors (ICIs) is approved in the first line therapy of advanced non-small-cell lung cancer. To date only PD-L1 expression on tumor cells has been found to be a marker of response to ICIs. The efficacy of ICIs as well as the susceptibility to immune-related adverse events are highly individual, so immune biomarkers are widely investigated. The candidates for predictive factors for ICIs immunotherapy include cancer cell antigenicity, presence of regulatory/suppressory molecules on cancer cells, cancer stem cells or on exosomes, and, on the other hand, an immune status of the patient. Cancers with high immune infiltration in the tumor milieu, referred to as “hot” tumors, seem to ensure a better response to ICIs than the “cold” ones. BALF analysis may replace cancer tissue examination, which is of limited access in advanced stages, for the recognition of the nature of immune response in the tumor environment. Tumor mutational burden (TMB) was shown to correlate with a good response to ICIs, especially when combined with other anticancer therapies. The present paper demonstrates the results of recent studies on lung cancer characteristics which bring us closer to the definition of useful prognostic/predictive factors.

## Introduction

The role of pathology in medicine is of great importance and is basic in diagnostic procedures of almost all diseases. Unfortunately, it is historically associated with such classic methods like autopsy and light microscopy. In the era of progress of molecular methods and new techniques pathology might seem a little obsolete. Nothing more confusing. The basic tissue examination is the first step leading to a deeper diagnosis. It is applied in almost all medical disciplines but in oncology it is especially noticeable. Modifications of pathological classification of different tumors are not rare as every few years new rules of classification are introduced. This is due to progress in pathological methods but the main reason is advancement in therapy. This can be compared to a closed circle: new therapies require a new classification, and once a new classification is possible,- a better selection to new therapies can be offered to patients.

Lung cancer has been the best example of such an important change in pathological classification strictly connected with therapeutic modalities over the last years. Lung cancer is leading in incidence and mortality, the estimated number of deaths worldwide is about 2 million per year (1). The insidious onset of disease, a lack of specific signs and symptoms and failure of screening methods result in a delayed diagnosis and a very large number of advanced stages at recognition (>70% of all cases). Lung cancer is an exceptional tumor also for other reasons. Tobacco smoking is a well-documented risk factor in oncology and, in spite of some efforts, remains an acceptable behavior in many countries. The influence of environmental agents other than tobacco smoke and comorbidities like chronic obstructive pulmonary disease (COPD), interstitial lung diseases (ILD) might result in the development of lung cancer in very special conditions in the respiratory tract.

The main histological classification into small-cell lung cancer (SCLC) and non-small-cell lung cancer (NSCLC) was the basis for treatment until the early 2000s (2). Currently a pathological report is not considered complete without a precise recognition of NSCLC subtypes. Nowadays two main NSCLC subtypes: adenocarcinoma (ADC) and squamous cell carcinoma (SQCC) are regarded as two different cancers requiring different therapeutic options (3). What differs these two types is, among others, their molecular pattern and susceptibility to different therapies. For ADC the tyrosine kinase inhibitors (TKI) were introduced in 2005 after the discovery of treatable oncogenic alterations with the first erlotinib, acting in the case of a confirmed mutation in the epidermal growth factor receptor (EGFR) gene. This targeted therapy resulted in the improvement in survival rates in advanced disease (2). The first large study Iressa Pan Asian Study (IPASS) indicated the predictive importance of EGFR mutation (4). And from this moment the new role of pathology has begun, with careful NSCLC subtypes diagnosis on the one hand, and the detection of molecular alteration by immunohistochemistry (IHC) or fluorescence *in situ* hybridization (FISH) and cooperation with molecular diagnostics, on the other hand. It should be noticed that pathological classification goes together with an upgraded clinical classification (5).

## Lung Cancer in Light Microscopy

The history of lung cancer classification has developed since the 1970s (6). The recent WHO classification of lung cancer is suitable for clinical practice and presents the possibility of correct recognition of cancer types in large specimens (e.g., surgical) as well in a small biopsy (e.g., cytology), It differs from the one published in 2004. The advantages of the 2015 classification are as follows:

- Application to small biopsy and cytological procedures.
- Description of IHC markers for a more precise classification of NSCLC.
- Addition of premalignant changes to the classification: early lesions of ADC and premalignant SQCC.
- Changes in the classification of adenocarcinoma (ADC).
- Genomic information for various types of lung cancers (7, 8).

**Figure 1:**
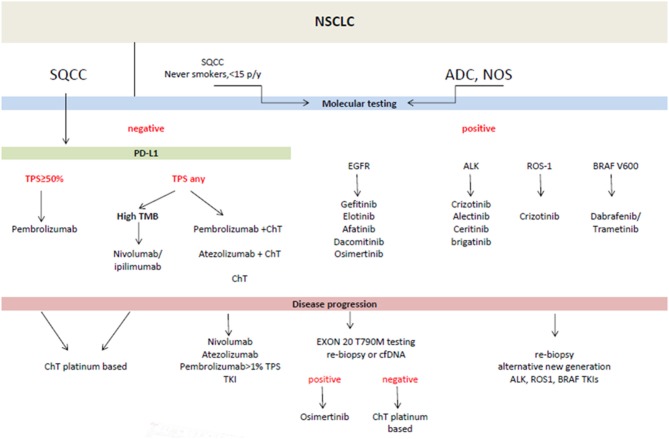
Treatment of advanced metastatic non-small cell lung cancer (NSCLC)- according to ESMO guidelines (10). ADC, adenocarcinoma; cfDNA, circulating free DNA; ChT, chemotherapy; NOS, not otherwise specified; PD-L1, programmed death ligand; SQCC, squamous cell carcinoma; TMB, tumor mutational burden; TKI, tyrosine kinase inhibitors; TPS, tumor proportion score.

**Figure 2:**
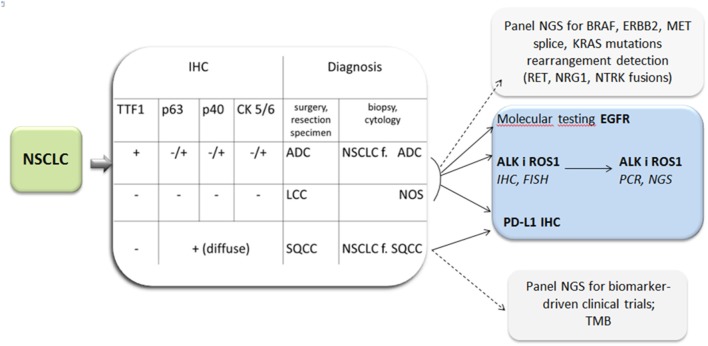
Lung cancer diagnosis: diagnosis by light microscopy with immunohistochemistry (IHC) and indication for molecular testing and PD-L1 expression tests. ADC, adenocarcinoma; f, favor; LCC, large cell carcinoma; NOS, not otherwise specified; SQCC, squamous cell carcinoma; TTF1, Thyroid transcription factor 1.

In practice the new classification is dedicated to <30% of lung tumors available for final diagnosis in surgical specimens and more than 70% in biopsy specimens. The former include a small biopsy and cytological materials. The development of cytopathology dates back to 1980 when fine needle aspiration (FNA) was introduced as an effective method of solid tumors diagnosis (9). Aspiration cytology replaced exfoliative cytology (sputum, bronchial washings) with evident prevalence. For many years cell smears were considered sufficient diagnostic material from needle aspirations on the basis of cell morphology. In lung cancer it enabled the pathologists to distinguish SCLC from NSCLC and it was satisfactory for oncologists. At that time two therapeutic options were in use: surgical treatment vs. radio-chemotherapy in advanced stages of cancer. Nowadays the therapy of lung cancer is more sophisticated, almost individually tailored (Figure 1). To meet the requirements of current histological classification an adequate number of cells is needed. It is essential for IHC and the confirmation of ADC (or non-squamous type) and for further molecular testing. Thus, a cell block technique was elaborated (11). The diagnosis of NSCLC in a small biopsy is limited to ADC, SQCC, and not otherwise specified (NOS) type in the recent histological classification (Figure 2). On the other hand, this classification clarifies what extract should be delivered from a small biopsy and cytological samples (7).

ADC is in the center of attention for several reasons: the proportion of this type among NSCLC is increasing, up to 60% of NSCLC cases, ADC is less connected with smoking than SQCC, it is prevalent in women, more complex in morphology and genetics than SQCC (12). A highly specific marker for ADC is Thyroid transcription factor 1 (TTF1). The detection of the expression of TTF1 confirms ADC and is useful for differential diagnosis between ADC and SQCC, especially in low differentiated cancers. Additionally, this marker helps distinguish primary lung ADC from adenocarcinoma of other body sites (13). The TTF1 positivity is also observed in SCLC and obviously in thyroid carcinoma. This transcription factor is involved in morphogenesis, differentiation, and surfactant production of normal lung epithelial cells and it was presented as a factor of prognostic significance (14–16). TTF1 negative ADC were found to present low frequency of driver mutations and unfavorable prognosis (17). The other marker for ADC is CDX-2 in cases of primary lung ADC with “enteric phenotype” (18, 19). The histological classification of ADC was elaborated by specialists in 2011 (12). It must be mentioned that the former ADC subtype recently excluded from the classification, namely bronchoalveolar carcinoma (BAC) is still used by some clinicians and some authors. BAC was defined as a tumor of lepidic pattern without invasion, mucinous and non-mucinous subtypes with solitary as well as multifocal lesions. These forms of BAC could meet the criteria of preinvasive lesions (AAH) as well as minimally invasive adenocarcinoma and invasive mucinous ADC (20).

The recent ADC classification presents the forms of different degree of aggressiveness. The early forms of ADC could be detected in high-resolution computed tomography (HRCT) as ground glass opacities (GGO). The GGO identified incidentally should not be disregarded, it needs attention and long-term observation up to 5 years. The solid part visible indicates a possible malignant component. Accordingly to guidelines from the Fleischner Society in 2017 these nodules are categorized as either pure ground glass nodules or part-solid nodules, having both GGO and solid components on thin-section CT (21). In the 8th clinical classification of lung cancer the following description is used: for cT1mi (minimal invasive ADC)- solid part 0 <5 mm; for cT1a- solid part 6–10 mm; for cT1b- solid part 11–20 mm; and for cT1c- solid part 21–30 mm (22). In the study of Ting Ye et al. a large collection of about 1,000 tumors was analyzed (23). The study aimed at a clinico-pathological comparison of part-solid nodules (PSNs) with pure ground glass nodules (GGN) and solid nodules. The prognosis of PSNs differed significantly from the other forms. Interestingly, in all forms presented in HRCT the different subtypes of ADC were found, for example, an invasive ADC was present in pure GGN in 10% of cases.

The impact of adenocarcinoma subtypes on prognosis was widely investigated. The common observation was that poorest prognosis was observed in micropapillary and solid patterns of ADC. The 5 year disease free survival in one study was from 100% in minimal invasive ADC, by 69.7% in acinar, 66.7% in papillary to 43.3% in solid, and 0% in micropapillary ADC (24). In the recently published study the recurrence hazard in early stages of ADC was found to be higher in tumors with the presence of micropapillary or solid components (25). These findings are consistent with other studies. The microscopic pattern of ADC is accompanied by differences in molecular pattern. EGFR mutations are observed mainly in non-smoker females with adenocarcinoma showing a lepidic growing pattern and also in atypical adenomatous hyperplasia (AAH) of the lung (24, 26). BRAF v 600E mutation is associated with female sex, a never smoking status and presence of micropapillary features. Adenocarcinomas with rearrangement of ALK as well as ROS1, and RET genes share similar histologic features, such as solid signet-ring cells and cribriform formation (27).

The position of modern pathology in oncology has become more prominent in the twenty-first century, as the aim of pathological diagnosis is to help resolve the question who will be the recipient of new therapies and who will not. Not only prognostic but also predictive markers play a crucial role in planning an individual therapy. The predictive marker indicates the presence or lack of response to a particular therapy. This response is defined by commonly used end points in clinical trials. The predictive factor indicates the efficacy of treatment guided by this marker. It is recommended that “*a targeted therapy could be used when patient's tumor cells have a specific biomarker that predicts an effective response to the targeted therapy.”* (28). Thus, it is advisable that a pathologist should be a member of a multidisciplinary team (MDT). A MDT guarantees relevant treatment options and an individual treatment plan for each patient, which is an obligatory form of personalized medicine (29).

The diagnostic tumor samples are of crucial importance because as always “tissue is the issue.” There is a need for sufficient cancer cells for classic morphological diagnosis in hematoxylin-eosin staining, for IHC and for further molecular diagnosis. In addition, tumor and tumor-adjacent stroma could be the source of necessary information concerning biomarkers for immunotherapy.

**Table:** 

For diagnosis- tumor
For molecular testing- tumor
For biomarkers for immunotherapy- tumor plus stroma

Of course the more biopsy material is gained the better, and it is always good to preserve the redundant material for additional testing.

FNA was found to be a valuable procedure for lung cancer diagnosis, as it has been mentioned above. In practice FNA is obtained by a transbronchial or transthoracic approach. For many years FNA cell smears were considered to be suitable to identify cancer cells, making it possible to distinguish SCLC and NSCLC. Currently, when IHC and molecular testing have become a necessary component of lung cancer diagnosis cell smears could not offer enough cells to define cancer characteristics. Thus, the cell block allows a pathologist to preserve cell pellet as an equivalent of “tissue” and prepare a larger number of slides. For a pathologist cell smears and cell blocks are complementary: in the cell smear the morphological features of malignant cells are well visible, in a cell block IHC could be performed. FNA is a low-invasive procedure, useful for the recognition of lung cancer metastases in many body organs such as mediastinal lymph nodes, superficial (e.g., supraclavicular) lymph nodes, the liver, adrenal lesions, metastases to soft tissue, etc. Gwozdz P et al. recently discussed the progress in pathological diagnosis, pointing to the usefulness of IHC staining with pan-cytokeratine antibodies to detect occult micrometastases of significant value for cancer prognosis (30). Due to the simplicity of the method lung cancer is very often recognized not on the basis of primary tumor examination but from metastasis. It must have some impact on cancer characteristics and particularly on its molecular pattern. Another example of a less invasive method of lung cancer investigation is liquid biopsy. It is discussed in the next section, as this kind of biopsy was developed for molecular testing.

## Molecular Testing

### Molecular Alterations, Toward Treatment

**Figure 3:**
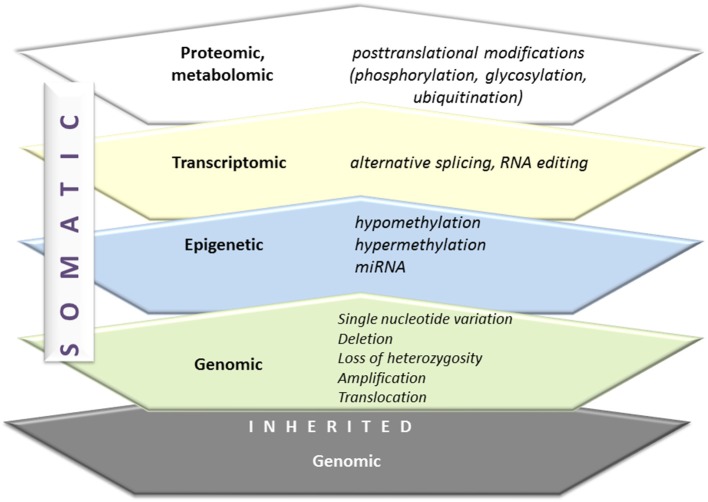
From genome to proteome. Inherited and somatic genomic alterations are additionally changed by epigenetic, transcriptomic, and finally, proteomic modifications. All of these phenomena are active in carcinogenesis.

The diversity of humans is a consequence of an inherited genetic signature as well as somatic genetic alterations. Figure 3 presents the spectrum of genetic alterations. Carcinogenesis in an individual is determined by these changes and additionally complemented by many new somatic mutations and epigenetic dysregulations. The Cancer Cell Line Encyclopedia illustrates the complexity of the spectrum of molecular changes in malignancy (31). Lung cancer and particularly NSCLC is a highly heterogenic tumor with a wide spectrum of somatic mutations. The mutated genes are involved in tumor biology with: the resistance to cell death, deregulation of metabolism, sustaining cellular proliferation, evading growth suppressors, enabling replicative immortality, activating invasion, and metastasis, inducing angiogenesis and inducing genomic instability and mutations (32). Among molecular alterations in lung cancer the potentially treatable ones are of special attention. These are mainly cancer-driving, “driver,” mutations, which means oncogenic mutations capable of triggering tumorigenesis (mutation in genes encoding signaling proteins, like tyrosine kinases, GTPases). The tumors are usually characterized by mutation in one gene, which facilitates therapeutic decisions (33). That one mutated gene is also responsible for the maintenance of cancer phenotype, a concept referred to as “oncogene addiction,” explained by Weinstein in 2002 in the article “Cancer. Addiction to Oncogenes–the Achilles Heel of Cancer” (34).

The description of the frequently mutated genes in lung cancer is presented in Table 1.

**Table 1:** Selected molecular alterations in non-small cell lung cancer with their subtypes, frequencies, and examples of targeted therapeutics /according to (32, 35, 36)/.

**Molecular alteration**	**Gene**	**Molecular subtypes**	**Methods of detection**	**Frequency (%)**	**Therapy**
Mutation	EGFR epidermal growth factor receptor	Exon 19 deletion and exon 21 L858	Direct sequencing, Real-time PCR, NGS	C 12-−27 EA 50–60	Gefitinib, Erlotinib, Afatinib, Osimertinib, Dacomitinib
		Exon 20 T790M		60	Osimertinib
		Exon 20 insertion		2.5	Poziotinib
	KRAS Kirsten rat sarcoma viral oncogene homolog	G12X, G13X		C-32 EA-2	MEK Inhibitors
	BRAF	V600E		2	Dabrafenib, Vemurafenib
	MET	Exon 14 splice mutation		3	Crizotinib, Cabozantinib, Capmatinib
	FGFR3	S249C		5.5	FGFR Inhibitors
	HER2	Exon 20		1	Afatinib, Trastuzumab, Dacomitinib
Translocation	ALK anaplastic lymphoma kinase	EML-4-ALK, TGT-ALK, KIF5B-ALK	FISH, IHC, NGS	5–7	Crizotinib, Ceritinib, Alectinib, Brigatinib, Lorlatinib
	ROS1 c-ros oncogene 1	CD74-ROS1, SLC34A2-ROS1, EXR- ROS1, SDC4-ROS1	FISH, NGS	3.4	Crizotinib, Ceritinib, Lorlatinib
	RET rearranged during transfection	CCDC6-RET, KIF5B-RET		1	Cabozantinib, Vandetanib, Lenvatinib, Alectinib, Ponatinib
	NTRK neurotropic tropomyosin receptor kinase	TPM3-NTRK, CD74-NTRK, MPRIP-NTRK		0.1	Entrectinib, Larotrectinib
	FGFR3 fibroblast growth factor receptor	FGFR3-TACC, BAG4-FGFR1		0.5–2	FGFR Inhibitors
Ampification	MET		IHC, NGS, FISH, real-time PCR	3–5	Crizotinib FGFR Inhibitors
	HER2		FISH, NGS, real-time PCR	13	Afatinib, Trastuzumab

*ADC, adenocarcinoma; C, Caucasian; EA, East Asians; FISH, fluorescence in situ hybridization; IHC, immunohistochemistry; NGS, next generation sequencing*.

The discovery of significant molecular alteration began in the 1980s and it was the mutation of KRAS- 1984, PTEN-1997, BRAF- 2002, EGFR, PIK3, HER2-2004, ALK, ROS1, MEK-2007 and more recently NTRK (33, 37). A frequency of driver mutations in Caucasians is as follows: AKT1 #1%, ALK 3–7%, BRAF 1–3%, EGFR 10–30%, FGFR1 20%, HER2 2–5% KRAS 15–30%, MEK1 1% NRAS 1%, PIK3CA 1–3% RET 1%, ROS1 1% (33). The frequency of EGFR mutations is higher in advanced recurrent ADC than in naïve cases before treatment (27 vs. 11%). The benefit of TKI and the value of EGFR mutation as a predictive marker was first documented for ADC. The map of molecular alterations in ADC is well-recognized and in this aspect there is a great discrepancy between ADC and SQCC (38, 39). A targeted therapy in its full sense is dedicated to ADC or in special conditions to non-squamous cell types (young, never smoking patients). The main treatable molecular abnormality in ADC is EGFR mutation and ALK relocation. What is more, activating mutations in KRAS, BRAF, ERBB2, and PIK3CA or translocations in RET and ROS1 occur (32). ADC cells also harbor the dysfunction of tumor suppressor genes with TP53, STK11, RB1, NF1, CDKN2A, SMARCA4, and KEAP1 (38). Different techniques are used for the deep characterization of DNA exome and genome in ADC. The sensitive next generation sequencing (NSG) was applied in the study of Imielinski et al. showing an example of efforts to describe a genetic “hallmark” defined by Hanahan and Weinberg (38).

### Conditions Interfering the Genome

Numerous environmental and intrinsic factors influence the genomic landscape of lung cancer (40). The main risk factor and the factor influencing lung cancer biology is tobacco smoke. It affects up to 80–90% of lung cancer patients. In addition, the importance of environmental tobacco smoke (ETS) must be appreciated, however, it is extremely difficult to measure. The smoking status is connected with higher mutational burden than never smoking (41). The results of the whole genome and transcriptome sequencing of NSCLC showed the differences in the genomic landscape between smokers and non-smokers. There were significantly higher mutation frequencies, KRAS, TP53, BRAF, JAK2, JAK3 in smokers, and EGFR mutations and ROS1 and ALK fusions in non-smokers.

Tobacco smoke seems to be responsible for high mutation burden in SQCC, similarly to other squamous cancers such head and neck SQCC. Campbell et al. analyzed molecular differences between ADC and SQCC (39). They found that non-synonymous mutation counts and neoepitope counts were not significantly different between ADC patients, which were smokers and patients with SQCC. The conclusion drawn from this complicated study was that: “*cancers arising from developmentally similar cells of origin across different tissues will be more similar than cancers arising from different cells of origin within an anatomically defined tissue”* *(**39**)*. The other concept of three lung units for tumorigenesis of different molecular basis was presented by Testa et al. These could be:

- Terminal alveolar unit (associated with EGFR mutations).
- Proximal inflammatory unit-squamoid (mutations of TP53).
- Proximal proliferative unit-magoid (KRAS mutations) (32).

In the recently published study Nahar et al. elucidate the dynamics of the genetic picture of ADC among Asian patients (42). They showed that smoking influences the enriched genetic milieu in ADC, while in never-smokers one truncal driver mutation is accompanied by a few co-drivers. This individual evolution of intratumoral mutations is possibly modulated by “mutations rates, driver nature, cytokine milieu, immune cell infiltration, and metabolic conditions.” This interesting direction of research into the changes in response to TKI was sketched in this study.

It is well-documented that sex and ethnicity could influence the differences in the genetic landscape. The problem of lung cancer among women is rising. On the one hand, the incidence among never smoking women seems to increase, on the other hand, women continue smoking and the reduction of the smoking habit is not so spectacular as among men (43–46). The differences between sex are strictly connected with the predominance of never-smokers among women and the predominance of ADC (46). Currently it is difficult to conclude that sex determines some specific molecular map of lung cancer, the problem is much more complex and needs deeper investigations. In smoking women the incidence of TP 53 mutations is higher than in men, a generally lower capacity of DNA repair is observed in women, which could be responsible for high susceptibility to smoking. A high rate of driver mutations was found in never smoking women with a higher rate in Asia and South America (46).

Two ethnic groups are usually compared in the context of lung cancer: Caucasian and East Asians (32, 35). The influence of ethnicity on the distribution of molecular alterations in lung cancer is well-documented and it concerns mainly ADC (47). EGFR mutation occurred in Asian population in 47.9% of adenocarcinoma cases, while in Western population in 19.2%. The frequency of EGFR mutations in lung cancer is the highest in Vietnam (64.2%), Taiwan (62.1%), and Thailand (53.8%). Driver gene mutations were detected in 79% of female never-smoker Asian patients with lung ADC and the most frequent was *EGFR* mutation (63%), whereas *KRAS* and *LKB1* mutations are more frequent in Western population.

Lung cancer is a malignant disease of old age. It is not frequent in young patients, but the prognosis is very poor. There are some differences in genetic alteration frequency and in younger patients the ALK and ROS1 rearrangements are observed (in addition with light smoking history) as well as HER2 mutation. Young lung cancer patients are those for whom molecular testing is dedicated sometimes independently of NSCLC type (32).

The circumstances presented above could result in the narrowing of the groups selected to the treatment. To date these differences have had no impact on the choice of therapy, but it seems evident that the effects of therapy and prognosis must be affected in some way.

Small-cell lung cancer (SCLC) is a distinct histological type of lung cancer (8). It is strictly connected with tobacco smoking. This fact and positivity to neuroendocrine markers cause the complexity of genetic aberrations with low specificity. The aggressiveness of SCLC could be explained by frequent inactivation of tumor suppressor genes and a lot of mutations, but not specific driver mutations. The classic mutations EGFR and KRAS are infrequent (48).

### Methodology of Molecular Testing

Molecular testing aiming at the recognition of genetic abnormalities in cancer and, mainly, at confirming or excluding the presence of treatable mutations should be performed in reference laboratories in cooperation with pathologists. Guidelines of the College of American Pathologists (CAP), the International Association for the Study of Lung Cancer (IASLC), and the Association for Molecular Pathology (AMP) were published in 2013, recommending “which lung cancer patients and samples should be tested, which genes should be tested, and how these tests should be designed, validated, and executed.” The reviewed version was published in 2018 (49). It is complemented by guidelines for non-adenocarcinoma, the use of IHC, NGS, liquid biopsy, tests for important recommendations and expert opinions from this document are as follows:

1. For new genes:
  - It is strongly recommended that ROS1 testing must be performed on all lung adenocarcinoma patients, irrespective of clinical characteristics.
  - In case of positive IHC for ROS1 it should be confirmed by molecular testing.
  - BRAF, RET, ERBB2 (HER2), KRAS, MET molecular tests are not recommended routinely, it is appropriate to include these genes mutation analysis as part of a larger testing panel performed either initially or when routine EGFR, ALK, and ROS1 testing are negative.
2. For methods:
  - In expert opinion multiplexed genetic sequencing panels are preferred over multiple single-gene tests to identify other treatment options beyond EGFR, ALK, and ROS1.
3. For other cancers than ADC:
  - Physicians may use molecular biomarker testing in tumors with histologies other than adenocarcinoma when clinical features indicate a higher probability of an oncogenic driver.
4. For testing:
  - Pathologists may use either cell blocks or other cytologic preparations as suitable specimens for lung cancer biomarker molecular testing.
  - Expert consensus opinion: laboratories should use, or have available at an external reference laboratory, clinical lung cancer biomarker molecular testing assays that are able to detect molecular alterations in specimens with as little as 20% cancer cells.
  - Strongly recommended testing for T790M: laboratories testing for EGFR T790M mutation in patients with secondary clinical resistance to EGFR- targeted kinase inhibitors should deploy assays capable of detecting EGFR T790M mutations in as little as 5% of viable cells.
  - For ALK rearrangement FISH and IHC techniques are equivalent, it is not recommended to repeat testing for ALK if progression after ALK TKI is observed.
5. For free DNA testing:
  - In some clinical settings in which tissue is limited and/or insufficient for molecular testing, physicians may use a cell-free plasma DNA assay to identify EGFR mutations.
  - There is currently insufficient evidence to support the use of circulating tumor cell molecular analysis for the diagnosis of primary lung adenocarcinoma.

### Liquid Biopsy

A liquid biopsy is a very special material in lung cancer diagnosis and management. The idea to use blood and body fluids for oncological investigations aims at the replacement of invasive diagnostic methods and a more efficient monitoring of disease progress and therapeutic efficacy (50, 51). Apart from peripheral blood (PB), pleural effusion, BAL fluid, an FNA cell suspension fulfills the criteria for liquid biopsy in case of lung cancer.

The use of plasma testing allows a patient to avoid invasive biopsy procedures. In case of an accurate diagnosis it could reduce the artifact introduced by formalin fixation of tissue specimens and tumor heterogeneity. However, one important fact should be kept in mind: changes in PB reflect not only cancer but all health conditions of a patient, for example, intrinsic homeostasis, comorbidities, influence of environmental agents (smoking), so the blood based results should be considered with caution.

The general goals for liquid biopsy are: early diagnosis of lung cancer, diagnosis of metastatic disease, monitoring of therapies, identification of targets for therapy, recognition of acquired resistance during therapy (50, 51). Therefore, it was named “ambrosia for researches.” The useful contents of liquid biopsy include circulating tumor cells, free cancer DNA and exosomes. However, after first enthusiastic reports some difficulties have arisen.

It is evident that the presence of circulating tumor cells in PB result from tumor spread and metastatic disease. Thus, it cannot be expected to be an important element of early diagnosis. They constitute an attractive material for research as they bear genetic information, which could prove useful in molecular diagnosis of difficult cases and in monitoring the molecular profile of cancer during targeted therapy. Detection systems of CTCs are different, some are based on the expression of EpCAM on these cells with different specificity and sensitivity (50). We used EpCAM to confirm the epithelial origin of putative lung cancer stem cells (CSCs) CD133+ in circulation. We found a small number of CSCs in the peripheral blood of lung cancer patients, which correlated with metastatic potential (52, 53).

The detection of abnormal genetic material from cancer cells and circulating free DNA (cfDNA) is sensitive and is now perceived as a molecular signature of cancer to identify mutations (54). It should be highlighted that cfDNA in PB is recommended for EGFR and T790M mutations (49). NGS was presented as the best way of cfDNA analysis. In a recent study by Zugazagoitia et al. NGS of cfDNA was performed. In this multicenter study patients resistant to TKI and different mutational status were included. The detectable actionable alterations of cfDNA were found in 55–60% of patients with resistance to first/second-generation TKIs, osimertinib and ALK/ROS1 TKI. It allowed the physicians to introduce appropriate molecular-guided therapies (55).

Exosomes are promising small structures bearing valuable information of cancer cell phenotype and molecular signature. These are small vesicles spontaneously released from different cells (56, 57). Tumor-derived exosomes are involved in many processes during carcinogenesis and progression as they carry cancer tissue specific information in proteins and miRNA. Among others they are capable of modifying the immune system thanks to the cargo of checkpoint molecules and cancer antigens (58). The development of the methods of exosomes analysis will lead to their application to clinics in the future.

## Lung Cancer Immunity

The success of immunotherapy with checkpoint inhibitors (ICIs) of solid tumors such as lung cancer has revolutionized our perception of cancer and its environment of development. The place of ICIs is established in the first line of advanced NSCLC and is moving toward neoadjuvant therapy, combined therapies, SCLC treatment (59, 60). In lung cancer PD-1/PD-L1 inhibitors were shown to be superior over chemotherapy (61), recently a durable effect of combination therapy with anti-PD-1 nivolumab and anti-CTLA-4 ipilimumab in advanced cancer was shown (62). In clinical trials as well as in real life not all patients benefit from ICIs and the response to treatment is individual. However, in some patients (about 20%) the prolonged survival (individually up to 7 years) even in metastatic disease is achieved. In contrast to molecular-guided targeted therapies there are no predictive markers for immunotherapy apart from the expression of PD-L1 on cancer cells. A better response to PD-1 blockers is observed in tumors expressing PD-L1. However, this is not explicit and we are still in the process of searching for an ideal biomarker for ICIs therapy, paraphrasing Hedge et al. the question is: “The Where, the When, and the How of Immune Monitoring for Cancer Immunotherapies” (63).

### PD-L1

PD-1 is a molecule expressed on immune cells, PD-L1 is a ligand for PD-1 overexpressed on cancer cells. The ligation of PD-1 with PD-L1 causes suppression of the function of cytotoxic lymphocytes (64). This scenario is simplified a little as both molecules could be detected on many other cells and another ligand PD-L2 exists. However, a new challenge for pathologists is the evaluation of PD-L1 on cancer cells aiming at a proper qualification of patients to immunotherapy. PD-L1 is detected by IHC in tumor samples. Adjusting an appropriate test and obtaining an appropriate number of tumor cells in specimens are two main complications linked to PD-L1 evaluation. The tests which were validated in clinical trials are still investigated.

In order to compare different IHC methods of PD-L1 expression evaluation the Blueprint PD-L1 IHC Assay Comparison Project was launched (65). Tumor samples were stained independently by Dako and Ventana reagents (total of four IHC assays from clinical trials were analyzed). The percentage of tumor cells exhibiting membrane PD-L1 expression is defined as tumor proportion score (TPS). A complicated analysis was performed to compare results reported by different pathologists. The results confirmed difficulties in the interpretation of IHC detection of PD-L1, concordance of three assays and indicated the necessity for further studies and observations in real life.

The multicenter international study EXPRESS was conducted to assess PD-L1 positive tumors across the world (66). Forty-five centers from 18 countries were involved. The samples of III/IV stage NSCLC were analyzed using the PD-L1 IHC 22C3 pharmDx kit (Agilent, Santa Clara, CA, USA). This large study once again showed the discrepancies in proportions of surgical vs. biopsy specimens in clinical practice. The tissue samples evaluated for PD-L1 expression (*n* = 2,368), 1,694 (72%) were obtained from tumor biopsies, whereas 610 (26%) from surgical resection. The distribution of PD-L1 was similar across the world: TPS ≥ 1% prevalence was found to be 52% in Europe, 53% in Asia-Pacific, 47% in the Americas, and 55% in other countries; and PD-L1 TPS ≥50% prevalence was 22% in Europe, 22% in Asia-Pacific, 21% in the Americas, and 24% in other countries. The second valuable evidence is that EGFR mutations and ALK translocation were accompanied by a lower frequency of PD-L1 positive tumors [TPS ≥ 50% were less common among patients with sensitizing *EGFR* mutations (13%) and those with *ALK* translocations (20%)].

The controversies concerning the examination of PD-L1 expression on cancer cells as a predictive factor are numerous (67, 68) and include:

- The aforementioned technical dilemma: quality of the test, slides reader skills.
- PD-L2 activity not detected.
- PD-L1 expression is not always related with immune activation.
- the response to treatment in negative tumors is observed in up to 20% of cases,
- PD-L1 is present not only on tumor cells.
- Cancer tissue is highly heterogenic, small biopsy (the only material for diagnosis) is obtained from different tumor areas.
- Expression of PD-L1 is dynamic, influenced by many factors, the main is IFNγ and connected with mutational burden.
- The function of ICIs is multipotent but we focus only on one pathway.

PD-L1 could be detected in materials other than primary tumor samples. In liquid biopsy PD-L1 is found on CTCs. Guibert et al. confirmed the presence of PD-L1 on CTCs by immunofluorescence nicely illustrating the presence of these two markers (69). They concluded that the evaluation of these cells is highly feasible. Unfortunately, no correlation between CTCs and tissue PD-L1 cells was found. No evidence for PD-L1 CTCs and response to nivolumab was found. An analysis of exosomes bearing PD-L1 seems to be another promising direction of research (58).

In our study PD-L1 was found on CSCs in needle aspiration guided by endobronchial ultrasound (EBUS TBNA) samples (52). The rationale for the identification of CSC or, according to some authors, “cells initiating tumor,” is that they seem to be more stable, guarantee resistance to systemic therapies and, in our view, are capable of bringing information for the modification of immune response in the site of the tumor. The study presented the phenotype of lung CSCs from different perspectives. Finally, the markers EpCAM, CD133, CD90, CD44, and CD184 were confirmed to be useful. To date we have identified these cells in the blood and metastatic lymph nodes (LNs). The material from EBUS TBNA was adapted for flow cytometry analysis. We detected these cells and found that their proportion is elevated in metastatic LNs. Next, the expression of PD-L1 on CSCs was evaluated. The PD-L1+ CSCs were also in higher proportion in metastatic LNs when compared to the free ones and were highest in ADC. Some correlation between suppressory immune cells in LNs and CSCs bearing PD-L1 was found in the study of Raniszewska et al. A higher PD-L1 CSCs were associated with disease progression/in press/. Yoshimura et al. in the study on 71 patients presented the evaluation of PD-L1 positive cells in EBUS-TBNA derived specimens by IHC and FISH. They found good concordance between PD-L1 detected in LNs and transbronchial biopsy (TBB), resected primary tumors, and resected metastatic tumors (70). Thus, these results confirmed the usefulness of EBUS/TBNA material, a typical method in lung cancer diagnosis, for the studies of local immune alterations. Obviously, in the case of LNs the cells analyzed are not obtained from primary tumor but from metastases and the dedifferentiation of the cancer should be taken into account.

### TME

The goal of cancer immunotherapy is to improve host anticancer response. The immunology of neoplasms is highly complicated and lung cancer does not deviate from these circumstances. In malignancy the host anticancer immune response is ineffective, the prevalence of suppression of the immune system over its activation is observed. Malignant tumors escape immunity in many ways (71–73). Thus, the key role of immunotherapy is to activate the host immune system to recognize cancer as a target for an immune attack. However, to be able to improve something it should have this “something.” In other words the knowledge of the host immune system, most notably at the site of primary cancer development, seems to meet the role of a biomarker in the broad meaning of this word. Thus, recently many studies have been conducted to better understand the character of the tumor microenvironment (TME). The importance of TME characteristics was shown in resected specimens of melanoma, colon carcinoma, breast cancer (74, 75). In lung cancer the availability of the whole tumor with its surrounding is limited, the resection rate is <30% and there is a tendency to collect small samples in diagnostics. The reservations concerning the evaluation of TME using classic microscopy methods are similar as in the case of PD-L1. The assessment of the immune cells in tissue samples is not well-standardized. Different methods applied do not yield comparable results; the “high” vs. “low” cell number, proportion, expression of the marker is rather subjective. For example, in our studies extrapolation from a quantitative analysis of cell smears was used and cells proportion was counted (76). The other researches apply different methods and reports from results (77, 78). Knowing the difficulties in the accessibility of representative tumor samples our proposal is to draw the knowledge on TME character from an analysis of BALF, which is possible to obtain during diagnostic bronchoscopy (79–81) and has many advantages such as:

1. Possible to perform in all stages of lung cancer, also advanced.
2. Is well-standardized.
3. Is reproducible, could serve to management of cancer progression and results of therapy.
4. In immune oncology is informative for:
  - Primary local immune status.
  - Modulation of immune response by treatment.
  - Differential diagnosis of immune related adverse events.

Our studies presented important differences in BALF inflammatory cell composition between patients with lung cancer and healthy subjects and between the lung affected by cancer and contralateral “healthy” lung and peripheral blood (82–86).

The studies showed that a rich lymphocytic infiltration in resected NSCLC is a good prognostic factor for survival (87). Today it is noticeable that tumor infiltrating lymphocytes (TIL) are heterogeneous and that “good” and “bad” cells are present among lymphocytes. These “good” cells are cytotoxic T cells, mainly CD8+ lymphocytes and in many studies it was shown that these cells are a good prognostic marker (88). CD8+ rich infiltrations in resected tumors were associated with better overall survival. In one study a high proportion of CD8+ cells with a low proportion of cells with the expression of the transcription factor Foxp3 was found to be a good prognostic marker (78). Foxp3 is a marker of regulatory T cells (Tregs) and an independent poor prognostic factor in malignancy (89). The CTLA-4 molecule is included in the checkpoint family of strong suppressive function (90, 91). Two forms of CTLA-4: superficial and intracellular were detected on the BALF Tregs in our study. A higher proportion of CTLA-4 was found in BALF from the lung with lung cancer when compared with the opposite “healthy” lung and peripheral blood (85). In a recent study we found elevated CTL-4 positive lymphocytes in regard to their maturation state (92). Lung cancer environment reflected by BALF was enriched with CTLA-4 positive maturated lymphocytes. The study by Paulsen and Donnem showed a complex role of CTLA-4 in tumor tissue and TME (91). This molecule was evaluated by IHC in resected NSCLC and the authors tried to assess their expression as a prognostic marker. The results were ambiguous, the presence of LNs CTLA-4 positive cells were shown to be a poor prognostic factor due to the stromal expression of this molecule positive in SQCC.

The meta-analysis by Soo et al. offered a summary of evidence for prognostic significance of immune cells in TME (93). The results are presented from cell to cell: from dendritic cells (DCs), macrophages, lymphocytes subpopulations, and checkpoint molecules on the cells. Ninety-six individual studies, assessing 21,752 cases were discussed. The analysis confirmed that DCs, NK cells, M1 macrophages, CD8+ T cells, and B cells in the tumor and stroma are associated with an improved prognosis, but stromal M2 macrophages, Tregs, and PD-L1 overexpression are associated with an unfavorable prognosis in NSCLC. As shown in our studies, all of the above-mentioned cells are feasible to identify in BALF from the alveolar space adjacent to the tumor (94). Recently we present elevated proportion of PD-1 and CTLA-4 positive cells and expression of these molecules on memory- activated CD8 cells in lung cancer milieu (92). However, the lack of confirmation of predictive value of the findings is the limitation of these studies.

In regard to TME as a predictive marker for ICIs therapy the general message is that rich immune infiltration determines a better response. The observations from other tumors are relevant in case of lung cancer. The inflamed, “hot” tumors are those in which PD-L1 as well as pro- and anticancer immune cells and mediators are present in high amounts, so in which the immune game “goes on” (95–97). The study presented by Chan-Young Ock was performed to confirm on a genetic level the concept that PD-L1 and CD8 rich tumors are those in which PD-1-PD-L1 blockade is effective (98). Indeed, the authors used the Cancer Genome Atlas (TCGA) to assess mRNA expression level of PD-L1 and CD8A, confirmed that PD-L1 amplification is associated with active tumor microenvironment immune type I. It was connected with high tumor mutational burden (TMB).

The effectiveness of immunotherapy in patients with mutated tumors with treatable driver mutations are of interest. It was shown that patients with EGFR mutations did not benefit from ICIs in overall survival vs. chemotherapy (99). Other studies confirmed this observation. As it was presented by Nahar et al. there are tumors with leading genetic alteration and the others with “genetic storm” (42). A high TMB showed an association with the benefit of ICIs in NSCLC, interestingly without correlation with PD-L1 (60). A high TMB was suspected to be a better predictive factor than PD-L1 expression for a durable clinical benefit (100). Rosenthal et al. presented the complexity of the genetic background of cancer immunity. They showed that the relationship between tumor antigens and the activation of the immune system are highly individual not only between subjects but also across the tumor (101). Recently Assoun et al. performed a molecular analysis in patients with advanced NSCLC treated with ICIs (mono- and combined therapy in first or second line). Genes were analyzed by NGS. TP53 gene mutations in advanced NSCLC patients treated with ICIs were found to correlate with better OS, PFS, and ORR, in comparison with TP53-wild-type patients (102). T53 mutations were significantly associated with smoking, the more T53 mutations correlated with PD-L1 expression. This study presented real life extended use of modern molecular pathology accompanied by PD-L1 evaluation and ICIs treatment.

The report of the Society for Immunotherapy of Cancer (SITC) was initiated to mark out the direction of research for contributors to cancer immune responsiveness (103). The reason for launching this project was the observation that “immune active cancers display a distinct genetic profile characterized by a high mutational burden.” The task force will elaborate the following topics: germline and somatic genetics, transcriptional changes, Immunogenic Cell Death (ICD), and experimental models of the immune landscape of cancer. ICD perceived as a strong immunogenic activator seems to be particularly interesting (104).

**Figure 4:**
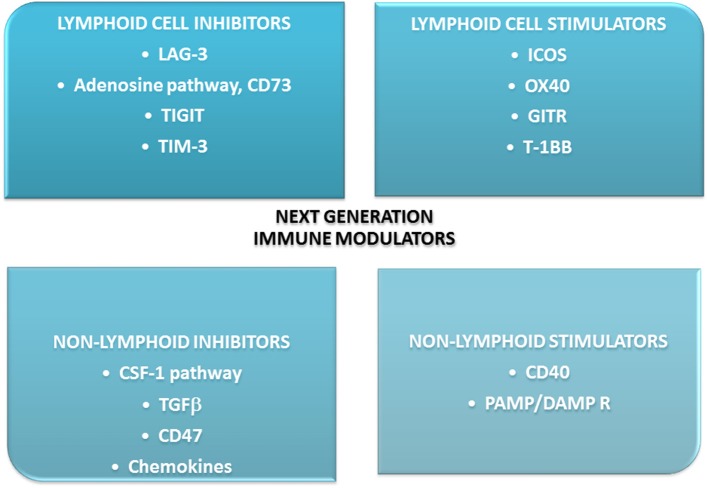
New immunomodulators in clinical trials- main directions of action. Most of them are used and effective in combination with immune checkpoint inhibitors: anti-PD-L1/PD-1 or anti-CTLA-4. 4-1BB, checkpoint co-stimulator; CSF-1, colony-stimulating factor-1; GITR, glucocorticoid-induced TNFR-related protein; ICOS, inducible co-stimulator; IDO, indoleamine-2,3-dioxygenaze/enzyme/; LAG-3, lymphocyte activation gene-3; OX-40, secondary co-stimulatory immune checkpoint molecule; PAMP/DAMP R, pathogen-associated molecular patterns/damage associated molecular patterns receptor; TGFβ, transforming growth factor; TIGIT, immune receptor, T cell immunoglobulin and ITIM domain; TIM-3, transmembrane immunoglobulin and mucin domain 3.

An analysis of the mechanisms of resistance to ICIs presented by Nowicki et al. might help understand the conditions in which ICIs work. The authors performed a deep analysis of possible reasons for this resistance. They mentioned the following conditions: lack of immunogenicity, enhanced T cell exclusion, lack of response to IFNγ, prevalence of immunosuppression in TME, upregulation of other immunosuppressive receptors on T cells: CTLA-4, T-cell immuno- globulin, and mucin domain-containing molecule 3 (TIM3), lymphocyte activation gene 3 (LAG-3), and V-domain Ig suppressor of T-cell activation (VISTA), low neoantigens production (105). Indeed there are more molecules on T cells capable of modifying their function (106). Some are activating, some suppressive. Kirilovsky et al. summarized data concerning possible immune cells and mediators as prognostic, predictive, and surrogate markers in ICIs therapy (107). Some of these factors were presented above and these are among others: PD-L1, TIL, CD8, CTLA-4, MDSCs. In this review some new agents are presented. These are checkpoint molecules, cytokines, chemokines, enzymes, as presented on Figure 4. One of them is indoleamine-2,3-dioxygenase-1(IDO-1), an enzyme involved in tryptophan catabolism. It was shown that inhibition of IDO-1 added to ICIs is effective in NSCLC. The more, evaluation of IDO-1 expression could be a promising biomarker for immunotherapy (108, 109). The new generation immune agents are widely investigated in many clinical trials. The significance of some new immunomodulators is that they are involved in similar pathways that “classic” checkpoints and are capable of potentiate ICIs (108, 110, 111).

### Pathologist Needed Immediately

A special type of adverse events is observed in immunotherapy with ICIs. These are immune non-infectious inflammation of different organs known as immune-related adverse events (irAE) (112). The pathogenesis of irAE is connected with autoimmunity. Checkpoint inhibitor pneumonitis (CIP) is a special example of irAE (113, 114). It is defined as: “new signs and symptoms from respiratory tract: dyspnea, cough, fever, pain, desaturation in exercise, in the presence of new infiltrations in chest X-ray without confirmation of infection” (114). The differential diagnosis includes: infection (new non-specific or opportunistic or re-activation of preexisting infection), tumor progression or pseudoprogression, dysfunction of other organs (heart, nervous system). The incidence of CIP is not very high but may be fatal in grade 4–5, when intensive treatment is needed and death occurs (113). An accurate diagnosis is crucial for an appropriate and rapid therapeutic decision. The treatment of choice includes systemic corticosteroids but continuation or discontinuation of immunotherapy also matters (115, 116). Since imaging methods are sometimes unspecific, a pathological diagnosis is decisive. Thus, CIP is a new challenge for pathologists. In our opinion, BALF could prove very useful in differential diagnosis of CIP as the following results are available from this low invasive, repeatable investigation: microbiological report, confirmation of exclusion of malignant cells infiltration, abnormal immune cell count suggestive for interstitial disorder. In our short experience the usefulness of BALF in CIP recognition was confirmed /personal observation/. In one study Tanaka et al. also supported the usefulness of BALF deep analysis in the evaluation of the character of CIP (117).

## Concluding Remarks

We have entered a new era of personalized lung cancer treatment, which needs careful diagnosis. The following essential conditions should be fulfilled before MDT decision concerning therapy: defined histological type of cancer, oncogenic alteration and PD-L1 expression on tumor cells. However, specific biomarkers are crucial to: anticipate natural progression of tumor, response to tailored therapies and resistance to treatment, predict immune-related adverse events. The participation of a pathologist in the treatment of a lung cancer patient care is indisputable. The complexity of lung cancer biology, of which this review is only a small excerpt, entitles us to put forward a hypothesis that apart from approved histological and clinical forms a range of lung cancer endotypes might exist. These could be: lung cancer in smokers, lung cancer and chronic lung diseases (COPD, ILD), mutated tumors, immune active tumors, sex- or ethnic-dependent cancer. All these require research and careful clinical observation to improve overall prognosis of lung cancer patients.

## Author Contributions

The author confirms being the sole contributor of this work and has approved it for publication.

### Conflict of Interest

The author declares that the research was conducted in the absence of any commercial or financial relationships that could be construed as a potential conflict of interest.
